# Placental Macrovascular Pattern from Pregnancies with Maternal Hypertensive and Fetal Growth Capacity Complications

**DOI:** 10.3390/pathophysiology31040050

**Published:** 2024-12-05

**Authors:** Kamilya Makhambetova, Yevgeniy Kamyshanskiy, Olga Ponamareva, Zhanna Amirbekova, Nazerke Oshakhtiyeva, Saule Kunanbaeva

**Affiliations:** 1 Institute of Life Sciences, Karaganda Medical University, Karaganda 100008, Kazakhstan; oshahtieva_n@mail.ru; 2 Institute of Pathology, Karaganda Medical University, Karaganda 100008, Kazakhstan; kamyshanskiy@qmu.kz; 3 Department of Biomedicine, Karaganda Medical University, Karaganda 100008, Kazakhstan; ponamareva@qmu.kz; 4 Department of Obstetrics and Gynecology, Karaganda Medical University, Karaganda 100008, Kazakhstan; amirbekovaz@kgmu.kz; 5 Department of Obstetrics and Gynecology, Astana Medical University, Astana 010000, Kazakhstan; nsg_85@mail.ru

**Keywords:** chorionic villi vessels, morphometric, placenta, fetal growth restriction, preeclampsia

## Abstract

Histomorphometric measurements of the wall thickness and internal diameter of the macrovessels of the chorionic villi of placentas from pregnancies complicated by preeclampsia or fetal growth restriction in comparison with normotensive pregnancy. Methods: The research included placentas from singleton pregnancies complicated by preeclampsia and/or fetal growth restriction, women delivered in medical institutions in Karaganda city (Kazakhstan). Placentas were divided into three groups: PE (*n* = 59), isolated FGR (*n* = 24), and PE with FGR (*n* = 41). The control group consisted of normotensive pregnancies, compared by gestation period. Placental examination and selection of placental tissue fragments were carried out in accordance with the consensus recommendations of the Amsterdam Placental Workshop Group. The sections were stained with hematoxylin and eosin and Masson trichrome. Morphometric measurements were performed using ImageJ software version 1.52p. Results: Our data showed that, in the PE group, there was a significant decrease in the wall thickness of the proximal and distal vessels with an increase in internal diameter compared with the control group (*p* < 0.01). In the PE + FGR group, there was a thickening of the wall of the proximal part of the vessels with a decrease in their lumen and a decrease in the wall thickness of the vessels with an increase in the lumen in the distal part compared with the control group (*p* < 0.01). Conclusions: Two histopatterns of placental macrovessels in preeclampsia were revealed: the histophenotype of diffuse (proximal and distal) ectatic macroangiopathy with a thin vascular wall with a decrease in the thickness of the muscle layer and the histophenotype of proximal fibromuscular sclerosis with vascular obliteration/spasm and distal ectatic macroangiopathy. We believe that significant structural differences in vascular remodeling may reflect the different temporal and spatial nature of the pathological factor. Future research is needed to investigate the associations between histopatterns of placental vascular remodeling in preeclampsia and long-term perinatal/maternal outcomes.

## 1. Introduction

Prolonged hemodynamic disorders in the «mother-placenta-fetus» system due to the action of various pathological factors can lead to remodeling of the vessels of the placenta and fetus, associated with long-term adverse effects on the cardiovascular system of the newborn [1,2,3,4] and the mother [5,6]. Structural changes in the placenta, leading to systemic disruption of blood circulation, decrease in perfusion, and oxidative stress, occur in a number of diseases in the mother, such as preeclampsia (PE) [7,8] or fetal growth restriction (FGR) [9,10]. Both of these pathologies are characterized by placental dysfunction and have a number of common pathological features, including disorders caused by pathological remodeling of the vascular wall.

PE is a common multisystem-specific pregnancy disorder accompanied by high blood pressure, fetal ischemia/hypoxia, and destruction of placental vessels [11]. Morphometric research on placental vessels has demonstrated various structural changes in the vascular tree of the placental villous tree in preeclampsia compared with normal pregnancy. At the same time, there are a number of discrepancies in the changes in the thickness of the vessel walls and the lumen of the vessels: along with reports of the thickening of the vessel walls due to smooth muscle hypertrophy or fibrosis and sclerosis, a number of authors report no changes in the vessel wall and even their thinning, in some cases, with an increase in the diameter of the vessels [12,13,14,15,16,17]. These contradictions emphasize the need for further research on changes in the placental vascular tree in preeclampsia using standardized morphometric analysis methods.

FGR is a condition of placental etiology in which the fetus does not reach the biological growth potential [18]. Placentas from pregnancies with FGR are more likely to have a reduced size and altered vasculogenesis and angiogenesis compared to placentas from physiological pregnancy, which are necessary to provide the growing fetus with nutrients. A number of research studies have reported vascular wall hyperplasia and lumen obliteration in FGR [19,20]. Other researchers have reported no differences in wall thickness and vascular diameter between FGR and physiological pregnancy [21]. Morphological changes in the blood vessels of chorionic villi in placentas from pregnancies complicated by PE or FGR are currently insufficiently researched. Histomorphometric assessment of placental vessels in PE and FGR may provide additional information to enable the understanding of the pathogenesis of these pathologies. 

The objective of this research was to perform histomorphometric measurements of macro vessels of chorionic villi of placentas from pregnancies complicated by PE and/or FGR in comparison with normotensive pregnancy.

## 2. Materials and Methods

### 2.1. Population Selection

A prospective cross-sectional case-control research was conducted in the pathology department of «Karaganda Medical University» NJSC from 1 January 2018 to 1 January 2023. The research included placentas from singleton pregnancies diagnosed with PE or FGR; the women delivered in medical organizations in Karaganda city (Kazakhstan). Patients were divided into three groups: PE (*n* = 59), isolated FGR (*n* = 24), and PE with FGR (*n* = 41). 

PE was defined as gestational hypertension of at least 140/90 mm Hg accompanied by one or more of the following newly identified conditions at 20 weeks of pregnancy or later: proteinuria, dysfunction of other organs of the mother (including liver, kidneys, neurological), or hematological disorders or uteroplacental dysfunction [22]. FGR was determined in accordance with the Delphi consensus definition [23,24].

All groups were compared by gestational age with the group of control pregnancies. The control group was defined as pregnancies without preeclampsia, gestational hypertension, or FGR, which ended with the birth of a healthy newborn during the research period with a complete medical record of the current pregnancy, whose placentas were sent for histological examination.

Exclusion criteria: rhesus incompatibility in pregnancy, HIV infection of the mother, SARS-CoV-2 infection during pregnancy, age under 18, multiple pregnancies, or congenital malformations of the fetus. 

The data obtained were depersonalized because each subject was encoded accordingly. All documented clinical data in the mother and pregnancy characteristics of the researched placentas are presented in Table 1. The comorbidity of the mother was determined by any concomitant presence of cardiovascular, endocrine, metabolic, autoimmune, and/or chronic kidney diseases. The pathology of the umbilical cord was defined as the presence of any of the following signs: attachment (marginal or vilamentous), length (long or short (for those cases when the absolute length of the umbilical cord measured during childbirth was known), the true node of the umbilical cord, single or multiple entanglements of the umbilical cord around parts of the fetus body. 

### 2.2. Placenta Specimen Examination

Placentas were examined in accordance with the standard protocol and consensus recommendations [25]. Placentas were sent for histological examination immediately after delivery if immediate examination of the placenta was impossible. They were stored in the refrigerator at 4 °C. Representative sections from the umbilical cord, membranes, and placental disc were taken for histological examination: 2 rolls of amniotic membranes, 2 fragments of the umbilical cord, 3 full-thickness sections of the placental parenchyma without macroscopic pathology were taken from the central two-thirds of the placental disc. 

Histological wiring of the native material and the manufacture of micro-preparations were carried out in accordance with the standard protocol of the pathology department of «Karaganda Medical University» as shown in previously published works [26,27].

### 2.3. Histomorphometrical Analyses

Placental villi were identified histologically in accordance with the Vogel M nomenclature [28]. The macro vessels of the stem villi were determined by the presence of a distinct middle shell represented by smooth muscle cells. 

All measurements were carried out manually by one researcher who did not know any information about the outcome of pregnancy and clinical history. Sections with no vessel lumen, poorly defined vessel wall boundaries, or obliquely cut arteries were excluded from the morphometric research. To determine the accuracy of the section, the ratio of the diameter of the vessel and the diameter perpendicular to it was calculated. Ratios greater than 1.3 were considered oblique slices and were excluded from the research.

Morphometric measurements were performed in the proximal and distal parts of the placenta. To determine these zones, two colored sections of the tissue of each placenta were divided into two zones of approximately equal width, located parallel to the basal plate: the parabasal layer adjacent to the basal plate (distal part); the parachorial layer adjacent to the chorial plate (proximal part). 

Morphometric measurements were performed in 20 arterioles of each placenta in each zone (proximal and distal). After calibration of the software, parameters including the thickness of the vascular wall and the inner diameter of the vessel were measured in each vessel using ImageJ.

To measure the thickness of the vessel wall, the outer and inner diameter of the vessel were measured on full cross sections. The thickness of the vascular wall was defined as half the difference between the outer and inner diameters of the vessel. For each placental vessel, four measurements were carried out in the directions corresponding to 12 and 6 o’clock, as well as 3 and 9 o’clock on the conventional dial, the arithmetic mean of four measurements for each vessel was taken as the result. These measurements were then averaged and recorded as the average vessel wall thickness for each of the placentas.

### 2.4. Ethics Statement

The research has been carried out in accordance with the Declaration of Helsinki (2013) of the World Medical Association [29]. The research was approved by the Ethics Committee of NJSC “Karaganda Medical University” (25 September 2024). Written informed consent was obtained from all patients before being included in the research.

### 2.5. Statistical Analysis

Statistical analysis was performed using SPSS (v.25.00, IBM Statistics, Armonk/New York, NY, USA). The differences between the groups were calculated using the Mann–Whitney U-test, the χ^2^ test adjusted for Yates continuity. The differences were considered statistically significant at *p* < 0.05.

## 3. Results

Demographic and clinical characteristics of the women along with the clinical characteristics of the pregnancies and newborns in the control group (normotensive pregnancy) and groups with preeclampsia and complications associated with impaired fetal growth are presented in Table 1.

The groups were comparable in terms of gravity, parity, and age of the mother and did not differ by the gender of the newborn and the time period before the placenta was received for histological examination. At the same time, women of PE and PE + FGR groups were more likely to suffer from chronic diseases in comparison with the control group.

### 3.1. Histomorphometric Measurements of Vessel Wall Thickness

**Figure 1:**
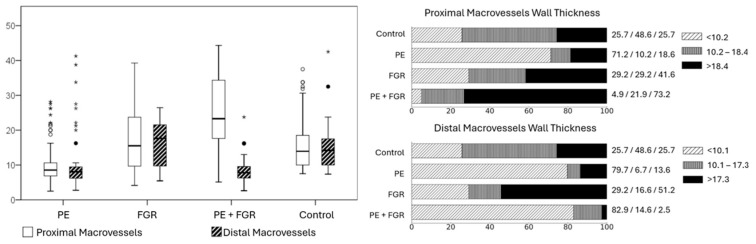
Wall thickness of proximal and distal macrovessels of placentas of the control group (normotensive pregnancy) and groups with preeclampsia and complications associated with impaired fetal growth, microns. Diagram illustrating the distribution of placental macrovascular wall thickness (μm) in the study groups (circles (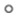) minor emissions, defined as values in the range from 1.5 to 3 interquartile ranges (IQR); stars (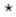)—extreme emissions, defined as values greater than 3 interquartile ranges (IQR).); Assessment of placental macrovascular wall thickness in the preeclampsia and fetal growth restriction groups compared with the control group.

The results of histomorphometric measurements on the wall thickness of the placental arterioles of the control group and groups with hypertension in the mother and complications associated with fetal growth restrictions are presented in Table 2 and in Figure 1.

In the control group, the average wall thickness of the macrovessels of the proximal part of the placenta was 15.7 ± 7.4 microns with a median of 13.9 microns and an interquartile range of 10.2–18.4 microns. In the distal part of the placenta, the average wall thickness of the macrovessels was 14.5 ± 5.9 microns, the median was 14.2 microns with an interquartile range of 10.1–17.3 microns. 

**Figure 2:**
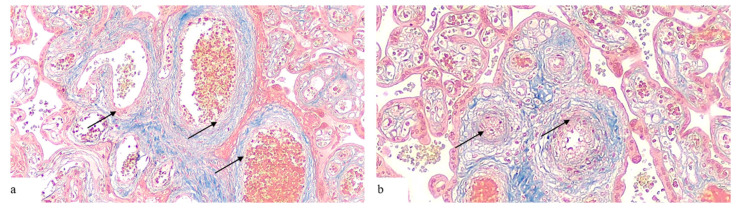
Histomorphological picture of vascular remodeling in the placentas of the researched groups. Masson trichrome, magnification: ×100. (**a**) PE, 36 weeks, ectasia of the vessel lumen with thinning of the muscle wall (collagen fibers are shown in blue, muscle tissue in red (arrows)); (**b**) PE + FGR, 36 weeks, thickening of the vessel walls with a decrease in the diameter of the vessel lumen.

In the PE group, the average value of the wall thickness of the proximal vessels was 10.7 ± 6.7 microns, of which, in 42 (71.2%) placentas, the average vessel thickness was less than 10.2 microns. In 6 (6.7%) placentas, it was in the range of 10.2–18.4 microns, in 11 (18.6%) placentas the average wall thickness of the macrovessels exceeded 18.4 microns. In the distal part of the placenta, the average wall thickness of the macrovessels was 10.4 ± 8.2 microns, of which, in 47 (79.7%) placentas, the average wall thickness of the macrovessels was less than 10.1 microns (Figure 2a). In 4 (6.7%) placentas, it was in the range of 10.1–17.3 microns, in 8 (13.6%) placentas it exceeded 17.3 microns.

In the FGR group, the average wall thickness of the proximal vessels was 17.2 ± 8.5 microns, of which, in 7 (29.2%) placentas, the average vessel thickness was less than 10.2 microns, in 7 (29.2%) placentas it was in the range of 10.2–18.4 microns, in 10 (41.6%) placentas the average wall thickness of the macrovessels exceeded 18.4 microns. In the distal part of the placenta, the average vascular wall thickness was 16.4 ± 6.6 microns, of which in 7 (29.2%) placentas the average vascular thickness was less than 10.1 microns, in 4 (16.6%) placentas it was in the range of 10.1–17.3 microns, in 13 (51.2%) placentas it exceeded 17.3 microns.

In the PE + FGR group, the average wall thickness of the proximal vessels was 24.9 ± 9.9 microns, of which in 2 (4.9%) placentas the average vascular thickness was less than 10.2 microns, in 9 (21.9%) placentas it was in the range of 10.2–18.4 microns, in 30 (73.2%) placentas it exceeded 18.4 microns. In the distal part of the placenta, the average wall thickness of the macrovessels was 8.5 ± 3.6 microns, of which in 34 (82.9%) placentas the average vessel thickness was less than 10.2 microns, in 6 (14.6%) placentas it was in the range of 10.1–17.3 microns, in 1 (2.4%) placenta it exceeded 17.3 microns (Figure 2b).

### 3.2. Histomorphometric Measurements of the Internal Diameter of Vessels

In the control group, the median of internal diameter of the vessels of the proximal placenta was 130.2 microns with an interquartile range of 97.9–154.4 microns, distal part 129.4 (82.3–156.4) microns (Table 2).

In the PE group, the median of internal diameter of the vessel lumen of the proximal placenta was 156.1 microns with an interquartile range of 115.2–188.5 microns, distal part 143.4 (104.9–192.9) microns.

In the FGR group, the median of internal diameter of the vessels of the proximal part of the placenta was 124.1 microns with an interquartile range of 100.8–149.5 microns, and the distal part of the placenta was 115.5 (105.8–130.4) microns.

In the PE + FGR group, the median of internal diameter of the vessels of the proximal part of the placenta was 100.8 microns with an interquartile range of 56.9–135.8 microns, and the distal part of the placenta was 161.6 (110.5–185.2) microns. 

## 4. Discussion

This research was devoted to histomorphometry of placental macrovessels from pregnancies with hypertension of mother and/or complications associated with impaired fetal growth ability in comparison with normotensive pregnancy. The obtained results of morphometric changes demonstrate significant variability in the thickness of the walls and lumen of the proximal and distal vessels of the placenta in preeclampsia, fetal growth restriction, and their combination.

Firstly, it was found that in the preeclampsia group, the walls of the proximal and distal macrovessels of the placenta were less thick in comparison with the normotensive pregnancy group (*p* < 0.01). These data are consistent with the results presented in research by other authors, who also note the thinning of the walls of the placental macrovessels in preeclampsia [13]. The revealed decrease in the relative thickness of the muscle layer in the macrovascular wall in preeclampsia confirms the hypothesis that preeclampsia leads to structural changes in blood vessels, impairing their ability to adapt against the background of increased vascular resistance, leading to a decrease in their elasticity and deterioration of perfusion. The observed ectatic macroangiopathy with vasodilation and thinning of the vascular wall may indicate a violation of the structure and tone of the vessels, affecting blood flow in the placenta and fetal nutrition. Diffuse involvement of both proximal and distal vascular sections indicates the systemic nature of the lesion, and a decrease in the relative thickness of the muscle layer in more than a third of the vessels indicates structural disorders that may be accompanied by a decrease in vascular tone, exacerbating circulatory disorders in the placenta.

Secondly, the results of morphometry allow us to distinguish two forms of preeclampsia depending on their vascular phenotype and association with the comorbidity of the mother. The first is primary preeclampsia, characterized by the diffuse presence of thin placental vessels with an ectated wall. This form of preeclampsia developed mainly in women without chronic diseases and was manifested by a more pronounced vascular lesion. The second is secondary preeclampsia, which is associated with the comorbidity of the mother, especially with diseases of the cardiovascular system. In this group, a more complex vascular histophenotype was observed in which the thickness of the walls of the distal vessels of the placenta could be thinner compared with the control group but remained thickened compared with the group of women with preeclampsia without chronic diseases (*p* < 0.05). This may be due to exposure to cardiovascular and metabolic risk factors of the mother, and reflects the prolonged or repeated exposure to a pathological factor, leading to obliteration of proximal vessels and compensatory dilation of distal vessels to maintain perfusion. The difference in vascular histophenotypes indicates the need for different approaches to preeclampsia, depending on the presence or absence of concomitant pathology of the mother. To confirm this assumption and uncover the mechanisms underlying these various forms of preeclampsia, further research on the effect of the comorbidity of the mother on vascular changes in the placenta is needed. 

Thirdly, the results of the research show the presence of changes in idiopathic intrauterine growth retardation, in particular, there is a tendency to increase the thickness of the walls of the proximal and distal vessels of the placenta. Although these changes do not reach statistical significance (*p* > 0.05), they may indicate hidden pathological processes associated with impaired vascular adaptation in FGR, which is consistent with the vascular changes in FGR described in the literature [20,30]. Trichromic staining by Masson showed that in the preeclampsia group with FGR, thickening of the walls of proximal placental vessels with fibromuscular sclerosis and deposition of collagen fibers were observed. These changes may reflect adaptive or compensatory reactions in conditions of chronic hypoxic stress.

The results of the research showed that in the placenta, the histophenotype of macrovessels with proximal fibromuscular sclerosis and distal ectatic macroangiopathy was associated with umbilical cord pathologies. Such changes indicate that the pathology of the umbilical cord can not only exacerbate the existing complications of pregnancy but also act as an independent risk factor for the remodeling of placental vessels, due to increased vascular resistance and decreased perfusion. 

The most important result of the research is the identification of two characteristic histopatterns of placental macrovessels in pregnancies with preeclampsia:

1. The histophenotype of diffuse (proximal and distal) ectatic macroangiopathy, characterized by an expansion of the lumen of both distal and proximal macrovessels of the placenta and accompanied by a relatively thin vascular wall with a decrease in the thickness of the muscle layer.
2. The histophenotype of proximal fibromuscular sclerosis with vascular obliteration/spasm and distal ectatic macroangiopathy.

We believe that the pattern of macrovascular pathology of the placenta is associated with the temporal and spatial nature of the action of the pathological factor: the action of the pathological factor at an early stage of development of the growing placenta leads to defective/incomplete formation of the muscle layer of the macrovessels, contributing to diffuse ectatic macroangiopathy. These morphological abnormalities can have serious physiological consequences for both placental and fetal function. We believe that the significantly lower wall thickness of the macrovessels is associated with adverse effects emanating from the primary pathological remodeling of the vessel wall and the detection of histopattern diffuse (proximal and distal) ectatic macroangiopathy in the placenta with preeclampsia can be considered an indirect marker of changes in the development of the cardiovascular system in a newborn. Research on this relationship is needed to predict the risk of cardiovascular diseases in adulthood in newborns born from pregnancy with this placental histophenotype, for their prevention and treatment in the future.

With the action of a pathological factor in a later period and/or prolonged or frequently repeated episodes of action, pathological processes have a cumulative effect on the vascular morphology of the placenta with the appearance of a synergistic effect of the «falling domino» type, when each subsequent pathological event overlaps and strengthens the previous ones. As a result, a combined vascular phenotype, including proximal fibromuscular sclerosis and distal ectatic macroangiopathy develops. We believe that this histophenotype not only reflects the severity of the pathological process in the placenta but also indicates long-term risks to the health of the mother.

## Figures and Tables

**Table 1:** Clinical characteristics of pregnancies for placentas studied.

Parameter		PE *n* = 59	FGR *n* = 24	PE + FGR *n* = 41	Control *n* = 70
Gravidity	Median	2	2	2	2
	25–75%	1.5–3	2–3	1–3	2–3
	Range	1–4	1–4	1–3	1–5
		*p* = 0.885	*p* = 0.223	*p* = 0.530	
Parity	Median	1	2	2	2
	25–75%	1–2	1–2	1–2	1–2
	Range	1–3	1–3	1–3	1–4
		*p* = 0.438	*p* = 0.601	*p* = 0.905	
Gestational age, (weeks)	Average	32.8	33.5	33.6	33.0
	SD	1.3	2.2	2.2	1.4
	Range	32–37	32–38	32–39	32–40
		*p* = 0.395	*p* = 0.506	*p* = 0.382	
Maternal age, (years)	Average	28.8	27.1	29.0	28.1
	SD	4.9	4.6	4.9	5.2
	Range	18–41	20–34	19–38	18–39
		*p* = 0.066	*p* = 0.597	*p* = 0.103	
Race, *n* (%)	Asian	47 (79.7)	17 (70.8)	30 (73.2)	50 (71.4)
	White	12 (20.3)	7 (29.2)	11 (26.8)	20 (28.6)
	Other	-	-	-	-
	Unknown	-	-	-	-
		*p* = 0.536	*p* = 0.956	*p* = 0.844	
Drugs, *n* (%)	Cigarettes	2 (3.4)	-	-	2 (2.9)
	Alcohol	-	-	-	-
	Other	-	-	-	-
	Unknown	4 (6.8)	-	2 (4.9)	2 (2.9)
		*p* = 0.561	-	*p* = 0.481	
Previous prenatal admission(s), *n* (%)	Yes	48 (81.4)	18 (75.0)	37 (90.2)	10 (14.3)
	No	11 (18.6)	6 (25.0)	4 (9.8)	60 (85.7)
	Unknown	-	-	-	-
		*p* = 0.001	*p* = 0.001	*p* = 0.001	
Delivery mode *n* (%)	Vaginal delivery	21 (35.6)	9 (37.5)	10 (24.4)	54 (77.1)
	Scheduled Cesarean delivery	26 (44.1)	10 (41.7)	20 (48.8)	10 (14.3)
	emergency Cesarean delivery	12 (20.3)	5 (20.8)	11 (26.8)	6 (8.6)
		*p* = 0.001	*p* = 0.002	*p* = 0.001	
Maternal oxygen given at delivery	Yes	43 (72.9)	17 (70.8)	35 (85.4)	30 (42.9)
	No	16 (27.1)	7 (29.2)	6 (14.6)	40 (57.1)
	Unknown	-	-	-	-
		*p* = 0.001	*p* = 0.019	*p* = 0.001	
Chronic diseases in pregnant women ^1^	Yes	34 (57.6)	0 (0)	41 (100)	11 (15.7)
	No	25 (42.4)	24 (100)	0	59 (84.3)
	Unknown	-	-	-	
		*p* = 0.001	*p* = 0.039	*p* = 0.001	
Placental weight (g)	Average	346.2	331.3	339.9	377.3
	SD	51.9	43.9	46.9	49.9
	Unknown	-	-	-	-
		*p* = 0.01	*p* = 0.001	*p* = 0.001	
The pathology of the umbilical cord	Yes	6 (10.2)	3 (12.5)	4 (9.8)	8 (11.4)
	No	53 (89.8)	21 (87.5)	37 (90.2)	62 (88.6)
	Unknown	-	-	-	-
		*p* = 0.819	*p* = 0.757	*p* = 0.946	
Baby’s sex, *n* (%)	Female	29 (49.2)	11 (45.8)	19 (46.3)	33 (47.1)
	Male	30 (50.8)	13 (54.2)	22 (53.7)	37 (52.9)
	Unknown	-	-	-	-
		*p* = 0.820	*p* = 0.912	*p* = 0.935	
Birth weight, gram	Average	2207.0	1708.1	1881.4	2391.0
	SD	326.5	153.6	271.1	217.4
	Unknown	-	-	-	-
		*p* = 0.001	*p* = 0.0001	*p* = 0.0001	
Delivery to processing (min)	Average	604.4	648.5	618.8	688.3
	SD	275.9	296.1	317.7	348.8
	Unknown	-	-	-	-
		*p* = 0.250	*p* = 0.541	*p* = 0.327	

^1^—diabetes mellitus, obesity 2–3 degrees, cardiovascular and autoimmune diseases. *p*-value reflecting statistically significant differences between the study groups and the control group.

**Table 2:** Morphometric measurements of the proximal and distal stem arteries of placental of the control group (normotensive pregnancy) and groups with preeclampsia and complication associated with fetal growth retardation, microns.

		PE *n* = 59	FGR *n* = 24	PE + FGR *n* = 41	Control *n* = 70
Proximal macrovessels					
Wall thickness	Median	8.4	15.5	23.3	13.9
	25–75%	6.9–10.7	9.9–23.2	17.6–34.3	10.2–18.4
	Range	2.5–28.1	4.1–39.3	5.1–44.3	7.5–37.5
		*p*_1_ = 0.001	*p*_1_ = 0.353	*p*_1_ = 0.001	
Lumen	Median	156.1	124.1	100.8	130.2
	25–75%	115.2–188.5	100.8–149.5	56.9–135.8	97.9–154.4
	Range	35.5–252.7	45.5–171.7	24.6–241.9	33.0–272.9
		*p*_1_ = 0.025	*p*_1_ = 0.138	*p*_1_ = 0.008	
Distal macrovessels					
Wall thickness	Median	8.1	17.7	7.8	14.2
	25–75%	6.3–9.5	9.9–20.9	6.3–9.5	10.1–17.3
	Range	2.7–41.3	5.4–26.5	2.6–23.8	7.4–42.5
		*p*_1_ = 0.001	*p*_1_ = 0.499	*p*_1_ = 0.467	
Lumen	Median	143.4	115.5	161.6	129.4
	25–75%	104.9–192.9	105.8–130.4	110.5–185.2	82.3–156.4
	Range	60.2–251.7	63.9–171.9	42.6–326.2	40.3–245.5
		*p*_1_ = 0.012	*p*_1_ = 0.504	*p*_1_ = 0.026	

*p*_1_ < 0.05, reflecting statistically significant differences between the study groups and the control group.

## Data Availability

The data supporting this study’s findings are available on request from the corresponding author.
